# Exposure to HIV prevention programmes associated with improved condom use and uptake of HIV testing by female sex workers in Nagaland, Northeast India

**DOI:** 10.1186/1471-2458-13-476

**Published:** 2013-05-15

**Authors:** Gregory Armstrong, Gajendra K Medhi, Michelle Kermode, Jagadish Mahanta, Prabuddhagopal Goswami, RS Paranjape

**Affiliations:** 1 Nossal Institute for Global Health, University of Melbourne, Victoria, Australia; 2 Regional Medical Research centre (RMRC), N.E.Region, Indian Council of Medical Research(ICMR), Dibrugarh, Assam, India; 3 FHI 360, New Delhi, India; 4 National AIDS Research Institute (NARI), Pune, India

## Abstract

**Background:**

There a concentrated HIV epidemic among female sex workers (FSWs) in the state of Nagaland, located in the north-east of India. Local non-government organisations (NGOs) are supported by the National State AIDS Control Society (NSACS) and the Avahan-funded Project ORCHID (Avahan is the India AIDS initiative of Bill & Melinda Gates Foundation in India) to deliver a range of interventions to FSWs including safe sex promotion, condom distribution, and testing and treatment of sexually transmitted infections (STIs). The commercial hub of Nagaland, Dimapur, is an important transportation node, and hosts a concentration of FSWs. This paper reports on comparative analysis of Integrated Behavioural and Biological Assessment (IBBA) data collected from FSWs in Dimapur in 2006 and 2009 to assess changes in condom use, HIV testing, and exposure to interventions.

**Methods:**

Two IBBA cross-sectional surveys were undertaken among FSWs in Dimapur in 2006 (Round 1) and 2009 (Round 2) using an interviewer-administered questionnaire and the collection of blood and urine samples. Respondent-driven sampling (RDS), a sampling technique for use among hidden populations, was used to recruit the samples.

**Results:**

When round 1 is compared with round 2, there was a marked and statistically significant improvement in the use of condoms at last sex with both occasional (35.2% to 72.4%) and regular (25.8% to 57.7%) clients, and an increase in the proportion having ever had an HIV test (8.9% to 29.1%). There was no evidence of an improvement in the proportional coverage of the HIV prevention services delivered to FSWs in Dimapur between round 1 and round 2. In round 2, FSWs exposed to the programme were more than twice (OR=2.27) as likely to consistently use condoms with occasional clients, four times (OR: 4.11) more likely to use condoms consistently with regular clients and nine times (OR: 9.08) more likely to have ever had an HIV test.

**Conclusions:**

We found evidence of an increase in condom use and HIV testing, and a strong and consistent association between programme exposure and condom use and HIV testing indicating that NGO HIV prevention programmes have been making a substantial contribution to HIV prevention among FSWs in Dimapur. However, there was no evidence of improved coverage of HIV prevention services, and there is a clear need to expand the reach of services in order for them to have an impact on a larger pool of FSWs.

## Background

Due to its large population, India is a major contributor to the South Asian HIV epidemic [1,2]. Six Indian states have consistently reported relatively high HIV prevalence: four in the south (Karnataka, Maharashtra, Andhra Pradesh and Tamil Nadu) and two in the northeast (Manipur and Nagaland). The state of Nagaland is located in northeast India, a geographically isolated region of the country characterised by a history of political unrest and under-development. The people of Nagaland are ethnically, culturally and linguistically distinct from the rest of India. Approximately 90% of Nagas (local residents) are Christian [3], and consequently, the Church is very influential in both the public and private spheres creating a socially conservative context in which public health issues are discussed and responded to [4]. Geographical isolation, fear of discrimination and lack of confidentiality are some of the barriers that inhibit access to standard health care services for groups at high risk of being infected with HIV such as female sex workers (FSWs).

Nagaland is consistently listed as one of the high HIV prevalence states in India [5]; adult HIV prevalence in 2009 was 0.78% compared with 0.31% nationally [6]. Based on prevention of parent to child transmission (PPTCT) testing data, Nagaland has the highest HIV prevalence among pregnant women in the country (0.89% compared with 0.19% nationally) [6]. Between 2003 and 2008 the HIV prevalence among female sex workers (FSWs) increased from 4.4% to 14.1% (compared with 4.9% nationally) [6,7]. The prevalence of STIs among sex workers is high, indicating that unsafe sex is occurring. For example, in 2009, 12.7% of FSWs in Dimapur, the commercial hub, had reactive syphilis serology (down from 22.1% in 2006) [8].

The HIV response in Nagaland is led by the Nagaland State AIDS Control Society (NSACS). In some districts the response is provided alongside government by the Avahan-funded Project ORCHID (Avahan is the India AIDS initiative of Bill & Melinda Gates Foundation in India). Most targeted HIV prevention services in Nagaland are implemented by non-government organisations (NGOs) funded by NSACS and/or Project ORCHID. The targeted interventions working with FSWs offer a range of services including safe sex promotion, condom distribution, and testing and treatment of sexually transmitted infections (STIs).

The HIV prevention services targeting FSWs in Dimapur, the commercial hub of Nagaland, have been primarily (although not exclusively) provided by Project ORCHID-funded NGOs since 2004. Dimapur is a commercial hub and an important transportation node that draws people from outside and inside Nagaland. It is the only town in Nagaland with an airport and a rail head. Dimapur hosts a concentration of FSWs; there are an estimated 1800 to 3500 FSWs in the city [9] who most commonly work in hotels (as opposed to more structured brothel-based sex-work), ‘booze joints’ (illegal bars), at home and on the street. While there are known hotspots for sex-work, there is no red light district as such. FSWs in Nagaland report substantial harassment from police, local gangsters and conservative religious and insurgent groups [10]. Consequently, from a public health perspective, these women remain hard to reach.

A major component of Avahan’s evaluation strategy for their HIV initiatives in India is the Integrated Behavioural and Biological Assessment (IBBA), comprising repeated cross-sectional surveys of target groups in selected districts. Two rounds of IBBA have been administered to FSWs in Dimapur, the commercial capital of Nagaland, in 2006 (Round 1) and 2009 (Round 2). Evidence for the effectiveness of HIV prevention programmes is essential given the importance of controlling HIV, and the considerable cost of the investment. Central to HIV prevention effectiveness is condom use and HIV testing among high-risk group members such as FSWs [11,12].

This paper reports on comparative analysis of Round 1 and Round 2 IBBA data in order to address the following questions in relation to FSWs in Dimapur: 1) Has condom use and the uptake of HIV testing improved? 2) Has programme coverage and intensity improved? 3) Are key vulnerable FSW sub-groups being reached by the programme? and 4) Is programme exposure associated with condom use and participation in HIV testing?

## Methods

### Study design

Two IBBA cross-sectional surveys were undertaken among FSWs in Dimapur in 2006 (Round 1) and 2009 (Round 2) using an interviewer-administered questionnaire and the collection of blood and urine samples. A detailed description of the IBBA objectives and methods are described elsewhere [8,13,14]. The study protocol was reviewed and approved by the Institutional Ethical Committees of Regional Medical Research Centre (RMRC), National AIDS Research Institute (NARI) and Family Health International (FHI).

### Sampling method

Respondent driven sampling (RDS) was used to recruit FSWs for participation in the IBBA surveys. RDS is a validated probability-based sampling method designed for use with hidden populations such as sex workers, injecting drug users, and men who have sex with men [15]. RDS is based on social network theory and builds on conventional snowball sampling, and has been used to recruit participants in several HIV biological and behavioural surveys [16]. RDS data are analysed using a statistical software package called RDSAT that generates weighted estimated proportions with confidence intervals [15,17]. The weights account for patterns of recruitment. For this study, a sample size of 400 was estimated based on an ability to detect changes in proportions of 15% at follow-up surveys from an assumed value of 50%, with 95% confidence and power of 90%. A design effect of 1.5 was applied to account for RDS methods [8,14,18].

### Data collection

Eligible participants were women aged 18 years or older, who had sex with men in exchange for cash or in kind payment at least once within the past one month. Demographic and behavioural data were collected using a structured, interviewer-administered questionnaire covering sex work history, condom use, drug use, treatment of STIs, and exposure to interventions.

Participants were requested to provide blood and urine samples for HIV and STI testing. Blood samples were tested for HIV and syphilis antibodies, and urine samples were tested for *N. gonorrhoea* (NG) and *C. trachomatis* (CT). Serum samples were tested for HIV by Microelisa (J.Mitra and Company, India), and positive tests were confirmed by Genedia HIV 1/2 ELISA 3.0 (Green Cross Life Science Corporation, South Korea). Serum samples were also tested for syphilis by rapid plasma reagin (RPR) test and confirmed by Treponema pallidum haemagglutination assay (TPHA). Urine samples were tested with nucleic acid amplification assay (Gen-Probe Aptima) for the detection of NG and CT. The questionnaire and the approach to testing the biological samples have been well-described elsewhere [8,14].

### Measures

#### Programme coverage

HIV prevention services for FSWs in Dimapur are primarily provided by Avahan-funded Project ORCHID NGOs, although a mix of other NGOs do provide services for FSWs. In our analysis, we included participant’s reports of contacts with either ORCHID or non-ORCHID NGOs.

To assess programme coverage we analysed responses to whether or not participants had accessed a range of HIV prevention services from NGOs, regardless of service provider. Coverage is defined as the proportion of FSWs in Dimapur who had accessed HIV prevention services. Participants were read a list of names of NGOs providing HIV prevention services in their area, and were asked if they had heard of any of them. If participants had not heard of any of the listed NGOs, it was assumed that they had not been exposed to any HIV prevention services.

There was a change in the wording of the questions related to exposure to interventions between round 1 and round 2. In round 1, the questions asking about contact with the programme were time bound, and asked with reference to the previous six months at a time when the program was only just being scaled up. In round 2, these questions were framed around having ever had contact with any of these services. Despite this difference we believe that, with caution, the two sets of responses are worthy of dissemination and comparison, particularly given it is the only data we have available that is linked with desired program outcomes such as condom use.

#### Programme exposure

Programme exposure was defined as having been exposed to at least one of the following three core programme services either in the last six months (Round 1) or ever (Round 2); 1) contacted by peer educators / staff of NGOs, 2) received condoms, and/or 3) visited the programme clinic. This approach to defining programme exposure is consistent with analyses of programme exposure among FSWs in other parts of India [19,20].

#### Programme reach to more vulnerable FSW sub-groups

Certain FSW sub-groups can be considered to be more vulnerable to HIV and STI infection, and therefore in particular need of the HIV prevention services offered by the programme. These sub-groups are young FSWs, FSWs new to sex work, FSWs with larger volumes of clients, FSWs with a history of drug use, FSWs without any source of income other than sex work, FSWs who had sold sex outside Dimapur, and those FSW who tested positive for an STI. It is important to monitor programme exposure among vulnerable FSW sub-groups given that factors such as younger age and a high client volume are indirect (distal) determinants of HIV risk [21].

### Data analysis

Descriptive analysis was undertaken using RDSAT (version 5.6) to generate proportion estimates with 95% confidence intervals adjusted for the recruitment patterns associated with RDS. P-values were calculated based on a test of interaction to compare changes in proportions in participant characteristics and intensity of programme contact [22]. Logistic regression analyses were conducted using SPSS version 19.0 to test the changes in condom use, HIV testing and programme coverage between round 1 and round 2 whilst adjusting for age, literacy, duration in sex work and client volume. Additional logistic regression analyses were conducted: 1) to estimate the strength of association between selected FSW characteristics and programme exposure using the recent round (round 2) data only, and 2) to estimate the strength of association between programme exposure and condom use and HIV testing in both round 1 and round 2; p-values were calculated based on a test of interaction to compare changes in the odds ratios between round 1 and round 2 [22]. All proportions and odds ratios produced in SPSS were done so using individualised weights generated for the respective dependent variables in RDSAT in order to adjust for the RDS sampling approach. For all logistic regression analyses, both unadjusted and adjusted odds ratios are presented along with their respective 95% confidence intervals.

## Results

### Participant characteristics

There were 426 FSWs recruited to participate in round 1 and 417 in round 2. Characteristics of the participants in both round 1 and round 2 surveys are presented in Table 1. In round 2, there were fewer participants aged 18–19 compared to round 1 (15.5% vs 22.4%), more participants aged 30–39 (26.2% vs 18.5%), and a higher proportion of participants who were illiterate (55.3% vs 38.7%). There were more participants who were in their first year of sex work in round 2 compared to round 1 (38.7% vs 19.4%), less participants who had 5 or more clients in the previous week (42.0% vs 52.4%), and more participants who had an income other than sex work (48.4% vs 38.4%). Fewer participants had consumed drugs in round 2 compared to round 1 (13.7% vs 20.3%), and in round 2, 13.7% of participants had sold sex outside Dimapur.

**Table 1:** Participant characteristics

	**2006 % (95% CI)**	**2009 % (95% CI)**	**p-value**
**Age**			
18-19	22.4 (17.8-26.3)	15.5 (11.1-19.5)	0.035
20-29	52.5 (47.8-57.5)	54.3 (48.9-59.7)	0.627
30-39	18.8 (15.1-23.0)	26.2 (22.3-31.1)	0.015
40+	6.3 (3.8-9.1)	3.9 (2.3-5.7)	0.136
**Literacy**			
No	38.7 (32.9-44.7)	55.3 (48.3-60.9)	<0.001
Yes	61.3 (55.4-67.1)	44.7 (39.1-51.7)	<0.001
**Duration in sex work**			
0-1 years	19.4 (15.8-23.6)	38.7 (32.7-43.2)	<0.001
2-4 years	42.8 (37.4-46.8)	42.1 (37.5-47.9)	0.846
5+ years	37.8 (33.4-43.2)	19.2 (15.5-23.5)	<0.001
**Volume of clients in previous week**			
Up to 4	47.6 (42.8-52.4)	58.0 (52.8-63.1)	0.004
5 or more	52.4 (47.6-57.2)	42.0 (36.9-47.2)	0.005
**Income other than sex work**			
No	61.6 (56.6-66.6)	51.6 (46.3-57.0)	0.009
Yes	38.4 (33.4-43.4)	48.4 (43.0-53.7)	0.008
**Ever consumed drugs**			
No	79.7 (75.4-83.3)	86.3 (82.0-90.1)	0.023
Yes	20.3 (16.7-24.6)	13.7 (9.9-18.0)	0.030
**Sold sex outside Dimapur**			
No	-- --	87.7 (83.6-91.4)	-- --
Yes	-- --	12.3 (8.7-16.4)	-- --

-- Data unavailable for analysis.

### Changes in condom use and HIV testing

There was a marked and statistically significant improvement in the use of condoms at last sex with both occasional (35.2% to 72.4%) and regular (25.8% to 57.7%) clients, and with main non-paying partners (13.4% to 36.4%) when round 1 is compared with round 2 (Table 2). Additionally, there was a significant increase in consistent condom use with both occasional (11.0% to 32.7%) and regular (5.5% to 19.7%) clients. FSWs were several times more likely to use condoms consistently in round 2 compared with round 1 with both occasional (OR=3.6) and regular clients (OR=4.3). Despite this increase, approximately two-thirds of FSWs in round 2 were still not consistently using condoms even with occasional clients. There was a significant reduction (55.8% to 43.7%) in the proportion of FSWs who, at least once during the previous month, had wanted to use a condom with a client but did not.

**Table 2:** Changes in condom use and HIV testing

	**2006 % (95% CI)**	**2009 % (95% CI)**	**Unadjusted OR (95% CI)**	**Adjusted OR**^**a **^**(95% CI)**
**Condom use with occasional clients**				
- last time	35.2 (31.8-41.4)	72.4 (65.0-78.8)	4.83*** (3.48-6.70)	4.97*** (3.47-7.12)
- every time	11.0 (8.7-15.3)	32.7 (25.8-39.5)	3.94*** (2.66-5.84)	3.58*** (2.36-5.43)
**Condom use with regular clients**				
- last time	25.8 (22.1-31.6)	57.7 (51.9-63.8)	3.93*** (2.93-5.29)	4.31*** (3.09-6.01)
- every time	5.5 (3.8-7.8)	19.7 (15.6-25.4)	4.22*** (2.59-6.88)	4.31*** (2.56-7.27)
**Condom use with main non-paying partner**				
- last time	13.4 (9.7 – 16.5)	36.4 (25.9-40.0)	3.71*** (2.53-5.42)	3.85*** (2.54-5.82)
**Wanted to use condom with client but did not at least once in past month**	55.8 (51.2-60.5)	43.7 (38.0-48.8)	0.62** (0.47-0.82)	0.73* (0.54-0.99)
**Ever had HIV test**	8.9 (6.6-12.3)	29.1 (25.1-35.5)	4.21*** (2.83-6.24)	4.32*** (2.82-6.62)

^a^ Adjusted for age, literacy, duration in sex work, and client volume. The odds ratios were generated using round as a predictor variable.

*p<0.05, ** p<0.01, *** p<0.001.

The proportion of FSWs having ever had an HIV test increased significantly between round 1 and round 2 (8.9% to 29.1%); FSWs were more than four times more likely to have had an HIV test in round 2 compared with round 1. However, many of the FSWs who tested HIV positive during the study reported never having had an HIV test previously, and thus were unaware of their positive HIV status. Encouragingly, the proportion of HIV positive FSWs who had never had an HIV test dropped from 80.8% in round 1 to 57.8% in round 2.

### Programme coverage and intensity

There was no evidence of an improvement in the proportional coverage of the HIV prevention services delivered to FSWs in Dimapur between round 1 and round 2 (Table 3). In round 1, approximately one-quarter (26.6%) had been contacted by a peer educator / NGO worker, rising marginally to 29.5% in round 2. There was no significant change in the proportion of FSWs who had received condoms (21.1% to 23.6%) or who had visited the programme clinic (24.1% to 21.9%). There were however significant increases in the estimated proportion of FSWs who had attended NGO meetings (2.2% to 14.4%) and seen a condom demonstration (18.2% to 26.2%); FSWs were more than one and a half times more likely to have seen a condom demonstration and almost 10 times more likely to have had attended NGO meetings in round 2 compared with round 1.

**Table 3:** Changes in programme coverage

	**2006 % (95% CI)**	**2009 % (95% CI)**	**Unadjusted OR (95% CI)**	**Adjusted OR**^**a **^**(95% CI)**
Contacted by peers / workers of NGOs	26.6 (22.4-32.6)	29.5 (24.1-35.3)	1.16 (0.86-1.56)	1.03 (0.74-1.43)
Received condoms	21.1 (17.6-27.2)	23.6 (19.4-29.8)	1.15 (0.83-1.60)	1.18 (0.83-1.68)
Seen a condom demonstration	18.2 (13.9-23.1)	26.2 (20.9-31.3)	1.59** (1.13-2.24)	1.70** (1.17-2.46)
Visited the programme clinic	24.1 (20.4-29.9)	21.9 (16.5-25.5)	0.88 (0.64-1.22)	0.80 (0.56-1.15)
Attended NGO meetings	2.2 (0.9-3.8)	14.4 (10.4-18.2)	7.54*** (3.56-15.97)	9.85*** (4.39-22.09)

^a^ The odds ratios were generated using round as a predictor variable and were adjusted for age, literacy, duration in sex work, and client volume.

*p<0.05, ** p<0.01, *** p<0.001.

Similarly, there was no evidence of an improvement in the intensity with which FSWs received services (Table 4). Among those who had been contacted by a peer educator / NGO worker, 30.8% had received more than five such contacts in the previous month in 2006, decreasing to 9.2% in round 2. There was no statistically significant change in the frequency with which FSWs were visiting the programme clinic to see a doctor. However, the majority (76.5%) of those who had obtained condoms from the programme reported that they had been receiving them at least once a week, indicating that many FSWs engaged with the programme were receiving condoms on a regular basis.

**Table 4:** **Changes in programme intensity **^**a**^

	**2006 % (95% CI)**	**2009 % (95% CI)**	**p-value**
Number of times contacted in the field by peers/workers in past month			
0	5.4 (1.7-11.7)	21.0 (11.1-25.9)	0.012
1-2	34.4 (23.6-51.0)	35.9 (25.4-45.5)	0.863
3-4	29.3 (15.9-38.1)	33.8 (28.5-51.2)	0.594
5+	30.8 (20.0-41.6)	9.2 (3.2-11.2)	0.001
Number of times visited the clinic to see doctor ^b^			
0	41.2 (27.5-53.7)	35.4 (22.7-48.9)	0.559
1	36.9 (27.9-53.2)	38.8 (27.7-50.8)	0.824
2	15.2 (5.2-20.1)	9.3 (4.3-15.3)	0.299
3+	6.7 (1.7-12.5)	16.6 (4.3-15.3)	0.133
Given condoms by peers/workers at least once a week	-- --	76.5 (66.6-84.3)	-- --

^a^ Among FSWs who had ever received these contacts.

^b^ Time period was during the last year in round 1, and during the last 6 months in round 2.

-- -- Data unavailable for analysis.

### Programme exposure among FSW sub-groups

Multivariate analysis of the data from round 2 revealed that some FSW sub-groups were less likely to have been exposed to the programme (Table 5). Just 17.2% of FSWs aged 18–19 years and 11.1% of those aged over 40 years had been exposed to the programme in comparison with over 30% of those aged 20–39 years. Compared to the youngest FSWs, those aged between 20 and 39 years were more than twice as likely to have been exposed to the programme. FSWs who had been engaged in sex work for more than 5 years were almost twice as likely to have been exposed to the programme in comparison to those who were in their first year of sex work. Those who tested positive for an STI (i.e. reactive syphilis serology, gonorrhoea, or chlamydia) were more than one and a half times more likely to have been in contact with programme services. Those who reported having sold sex outside of Dimapur were half as likely to have been exposed to the programme. No statistically significant difference in exposure was observed among other FSW sub-groups based on volume of clients in the previous week, having an income from activities other than sex work, and having a history of drug use.

**Table 5:** Logistic regression analyses of programme exposure by FSW sub-group, in 2009

**Characteristic**	**% exposed to programme**^**a**^	**Unadjusted OR (95% CI)**	**Adjusted OR**^**b **^**(95% CI)**
**Age**			
18-19	17.2%	1	1
20-29	33.0%	2.34* (1.16-4.74)	2.34* (1.13-4.85)
30-39	35.2%	2.57* (1.20-5.49)	2.79* (1.25-6.20)
40+	11.1%	0.70 (0.16-3.14)	0.59 (0.13-2.80)
**Duration in sex work**			
0-1 years	24.2%	1	1
2-4 years	31.3%	1.42 (0.86-2.34)	1.30 (0.78-2.17)
5+ years	38.5%	2.00* (1.11-3.59)	1.89* (1.04-3.45)
**Volume of clients in previous week**			
Up to 4	26.7%	1	1
5 or more	31.7%	1.27 (0.82-1.96)	1.31 (0.84-2.06)
**Income other than sex work**			
No	27.4%	1	1
Yes	31.8%	1.24 (0.81-1.89)	1.23 (0.79-1.91)
**Ever consumed drugs**			
No	29.2%	1	1
Yes	33.3%	1.24 (0.68-2.24)	1.24 (0.67-2.30)
**Sold sex outside Dimapur**			
No	31.3%	1	1
Yes	20.0%	0.53 (0.26-1.07)	0.44* (0.21-0.92)
**Any STI**^**c**^			
No	27.3%	1	1
Yes	37.5%	1.58 (0.99-2.51)	1.73* (1.07-2.80)

^a^ Proportion of FSWs who had been exposed to programme services (any one of; contacted by peers/workers of NGOs, received condoms, or visited the programme clinic), calculated in SPSS using weights generated in RDSAT.

^b^ Adjusted for literacy and place of solicitation.

^c^ Includes reactive syphilis serology, gonorrhoea, and chlamydia.

*p<0.05, ** p<0.01, *** p<0.001.

### Associations between programme exposure and condom use and HIV testing

Results of multivariate analysis supports an association between programme exposure and increased condom use and better uptake of HIV testing in both round 1 and round 2 (Table 6). Exposure to the programme was associated with condom use at last sex, and consistent condom use, with both occasional and regular clients in both round 1 and round 2. In round 2, FSWs exposed to the programme were more than twice as likely to consistently use condoms with occasional clients, and four times more likely to use condoms consistently with regular clients. The proportion of FSWs exposed to the programme who consistently used condoms with occasional clients rose from 17.0% in round 1 to 40.7% in round 2. There was no association between exposure to the programme and the use of a condom at last sex with main non-paying partners.

**Table 6:** Logistic regression analyses of condom use and HIV testing by programme exposure in 2006 and 2009

	**2006**			**2009**			**p-value**^**c**^
	**%**^**a**^	**Unadjusted OR (95% CI)**	**Adjusted OR**^**b **^**(95% CI)**	**%**^**a**^	**Unadjusted OR (95% CI)**	**Adjusted OR**^**b **^**(95% CI)**	
**Condom used at last sex with occasional client**							
Exposed							
No	26.7%	1	1	66.0%	1	1	
Yes	52.5%	3.03*** (1.98-4.63)	2.81*** (1.82-4.35)	79.4%	1.99* (1.16-3.39)	2.04* (1.11-3.74)	0.401
**Condoms used every time with occasional clients**							
Exposed							
No	7.8%	1	1	25.7%	1	1	
Yes	17.0%	2.41** (1.30-4.47)	2.01* (1.06-3.80)	40.7%	2.00** (1.21-3.28)	2.27** (1.28-4.02)	0.781
**Condom used at last sex with regular client**							
Exposed							
No	19.5%	1	1	50.2%	1	1	
Yes	38.7%	2.62*** (1.67-4.12)	2.30** (1.43-3.70)	69.9%	2.34*** (1.51-3.62)	2.05** (1.25-3.35)	0.742
**Condoms used every time with regular clients**							
Exposed							
No	3.2%	1	1	11.6%	1	1	
Yes	10.1%	3.33** (1.41-7.87)	2.73* (1.13-6.61)	33.1%	3.79*** (2.24-6.40)	4.11*** (2.28 – 7.38)	0.450
**Condom used at last sex with main non-paying partner**							
Exposed							
No	11.8%	1	1	33.0%	1	1	
Yes	16.9%	1.49 (0.81-2.73)	1.26 (0.67-2.40)	42.7%	1.52 (0.93-2.50)	1.29 (0.72-2.31)	0.957
**Had wanted to use a condom with a client but did not at least once in past month**							
Exposed							
No	57.3%	1	1	46.3%	1	1	
Yes	52.9%	0.84 (0.56-1.26)	0.83 (0.54-1.27)	36.2%	0.65* (0.43-0.97)	0.52** (0.33-0.83)	0.145
**Ever had HIV test**							
Exposed							
No	5.7%	1	1	12.9%	1	1	
Yes	15.4%	3.00** (1.52-5.93)	3.81*** (1.84-7.89)	57.1%	8.92*** (5.52-14.44)	9.08*** (5.34-15.44)	0.059

^a^ Proportion of FSWs using condoms or having ever had an HIV test, split between exposed versus not exposed to programme services, calculated in SPSS using weights generated in RDSAT.

^b^ Adjusted for age, literacy, duration in sex work, client volume, and place of solicitation.

^c^ P-values were calculated based on a test of interaction to compare changes between round 1 and round 2 in the odds ratios.

*p<0.05, ** p<0.01, *** p<0.001.

In round 1, around half of the FSWs reported that they had, on at least once occasion in the previous month, wanted to use a condom but did not. In round 2, the likelihood of this occurring was halved for FSWs exposed to the programme.

Those exposed to the programme were almost four times more likely to have had an HIV test in round 1, rising to 9 times more likely in round 2. There was a substantial increase between round 1 and round 2 in the proportion of FSWs exposed to the programme that had undergone an HIV test (15.4% to 57.1%), while the proportion of FSWs not exposed to the programme that had undergone an HIV test remained very low (5.7% to 12.9%). Furthermore, there was an increase in the proportion of HIV positive FSWs exposed to the programme who had previously undergone an HIV test from 20.0% in round 1 to 60.0% in round 2.

There were no statistically significant differences between the odds ratios in round 1 and round 2, although the difference in odds ratios with regard to HIV testing approached significance (p=0.059), suggesting that the association between programme exposure and both condom use and HIV testing largely remained constant.

## Discussion

This paper presents an assessment of the HIV prevention programme for FSWs in Dimapur, Nagaland, Northeast India using IBBA data from round 1 (2006) and round 2 (2009). The analysis provides evidence of a substantial increase in condom use and HIV testing between round 1 and round 2, and an association that links exposure to programme services with a greater likelihood of condom use and HIV testing reaffirming the importance of the harm reduction programme in reducing HIV risk behaviours. However, our analysis provides evidence that programme coverage remained far too low between round 1 and round 2 and that there is a pressing need to scale-up services. This suggests that the increases in condom use and HIV testing can only be partially attributed to the programme. We found that younger FSWs, those newer to sex work, and those who were more mobile were less likely to have been exposed to programme services.

There was a marked improvement in condom use at last sex between round 1 and round 2 with both occasional and regular types of clients, and with main non-paying partners. Reports of consistent condom use also increased. Despite this strong progress, the proportion of FSWs using condoms at last sex with commercial clients is considerably lower than that observed among FSWs elsewhere in India. For example, condom use with occasional clients is approaching, or exceeds, 90% in Karnataka (88.0%) [23], Mysore (89.9%) [24], Tamil Nadu (98.1%) [25], and Maharashtra (99.7%) [26]. Regardless of the higher condom use among FSWs elsewhere, condom use among FSWs in Dimapur has clearly improved substantially from a very low base.

There was an encouraging improvement in the uptake of HIV testing. Importantly, there was a decrease in the proportion of HIV positive FSWs who had never had an HIV test. Despite the improvement in HIV testing uptake among these FSWs, it is still a relatively and unacceptably low level of uptake. In comparison, IBBA data from elsewhere in India revealed that FSWs having undergone an HIV test ranged from 50.0% to 86.0% in Andhra Pradesh; 56.0% to 96.0% in Maharashtra; 60.0% to 77.0% in Karnataka; and 67.0% to 84.0% in Tamil Nadu [8].

We found no clear evidence of an improvement in programme coverage between round 1 and round 2. We did however find a consistent and strong association between programme exposure and both HIV testing and condom use with a range of partners in both rounds suggesting the programme was having a positive impact on those FSWs who had received services. The associations between programme exposure and both condom use and HIV testing were comparable to those in other parts of India where FSWs are also serviced by the Avahan-funded India AIDS Initiative [19,25,26]. Importantly, there was a very strong association between programme exposure and having undergone an HIV test. In round 2, those FSWs who were exposed to the programme were 9 times more likely to have had an HIV test; 57.1% of those exposed to the programme had undergone an HIV test compared to 12.9% of those who had not been exposed. The effect of programme exposure on HIV testing was pronounced among FSWs who tested positive for HIV; the proportion of HIV positive FSWs exposed to the programme who had previously undergone an HIV test increased markedly from 20.0% in round 1 to 60.0% in round 2.

The lack of progress with regard to programme coverage and the relatively lower use of condoms and uptake of HIV testing among FSWs in Dimapur, in comparison to FSWs from other parts of India, may be partly explained by the challenges associated with mobilising the local FSW community. Effective community mobilisation has been seen to be associated with improved condom use among other FSW populations [27]. However, sex work in Dimapur has historically been hidden with some known hotspots but few brothels and no red light district. FSWs fears of harassment from police, gangsters and conservative religious groups has meant that public disclosure as a sex worker is personally risky [10]. As a result, these women remain hard to reach and difficult to mobilise as a community, unlike FSWs in other parts of India [27,28]. The low, but increasing, rates of FSW attendance at NGO meetings seen in this study suggests that mobilising the FSW community in Dimapur will take more time and concerted effort.

Furthermore, previous research revealed considerable ethnic, cultural, religious and linguistic diversity among sex workers in Dimapur, with varying pathways to sex work, which can contribute to polarisation between local women and those from outside the state [10]. A high level of socio-cultural diversity may present a barrier to community mobilisation activities that seek to building unity and a sense of commonality within the FSW community in Dimapur.

Particular FSW sub-groups were more or less likely to have been exposed to the NGO programme interventions. FSWs who tested positive for an STI were more likely to have had contact with programme services. The higher exposure to programs among FSWs who had STI symptoms suggests that their exposure was related to seeking treatment for perceived STI symptoms, or that FSWs with an STI are riskier and more deeply engaged in sex work and thus more easily identified by programme services and more willing to use them. The study of Ketkesone et al. [29] in Laos suggests that FSWs prefer to seek treatment at drop-in-centres compared to public or private hospitals/clinics in order to maintain confidentiality. Subsequently, perhaps those who are exposed to programs due to perceived STI symptoms may also be exposed to other services such as HIV testing and condom distribution.

Younger FSWs (i.e. those under 20), those new to sex work, and those more mobile FSWs who had sold sex outside of Dimapur were all less likely to have been exposed to programme services. It is unsurprising that programme reach is lower among younger and newer FSWs, and this result has been found elsewhere in India [19]. They may be less likely to identify as sex workers, less connected to the sex work community, and less aware and/or trusting of NGOs, thus making them more hidden in their sex work and harder to identify and approach. Given their young age and that they are new to sex work, they may also have less knowledge of STIs/HIV and less skill and experience in negotiating a safer work environment including condom use.

Having sold sex outside of Dimapur could be interpreted as a proxy for being a more mobile sex worker, and/or having migrated from outside Dimapur; previous research on a sample of FSWs in Dimapur found that approximately 40% were born outside Nagaland. By definition, more mobile FSWs can be harder to identify and engage depending on the frequency and scope of their mobility. Analysis of data on FSWs from four high HIV prevalence states (Andhra Pradesh, Karnataka, Tamil Nadu and Maharashtra) identified that highly mobile FSWs (i.e. having sold sex in three or more places) were approximately three times more likely to be inconsistent in using condoms with either paying or non-paying partners [30], highlighting mobile FSWs as a key group of interest. FSWs who were born outside Nagaland may present additional challenges given there are likely to be barriers such as literacy in the local language and knowledge of local services that may be impeding access to services.

Overall, the results of our analyses highlight that NGO programmes in Dimapur are making a substantial contribution to the use of condoms and the uptake of HIV testing at least among the FSWs they are in contact with, including improved participation in HIV testing by HIV positive women, thereby facilitating their access to treatment services. However, coverage is far too low for effective HIV prevention, and there remains a clear and pressing need to scale-up services. This may require the development of interventions that specifically target the more difficult to reach groups such as the young and mobile FSWs. For example, younger FSWs who are new to sex work, and who do not yet identify with the FSW community, may be more likely to respond to younger peer educators, or more attracted to a less conspicuous HIV prevention service such as a beauty parlour that is strategically placed close to sex work hotspots, and that provides a range of services in addition to beauty services. The programme coverage needs to be broadened, without compromising quality, so that it can have the same impact on a larger pool of the FSWs working in Dimapur.

These study findings are subject to several limitations. The use of RDS methods to recruit participants creates limitations for multivariate analysis; guidelines for multivariate analysis of RDS data are still under development and require validation. Despite this limitation, RDSAT provides probability-based estimates of programme coverage, condom use and HIV testing, and weights were generated for multivariate analysis to best account for having used the RDS sampling methodology. Reporting of certain behaviours may have been influenced by recall and social desirability bias. As with all cross-sectional study designs, causal relationships cannot be firmly established.

Despite these limitations, the analysis in this paper presents results that have significant programming implications and improves our assessment of NGO-delivered HIV prevention services in Dimapur.

## Conclusions

The comparative analysis of FSW IBBA data from round 1 (2006) and round 2 (2009) presented in this paper indicates that there is evidence of an increase in condom use and HIV testing, particularly among FSWs exposed to HIV prevention services. We found strong and consistent associations between programme exposure and condom use and HIV testing indicating that NGO HIV prevention programmes have been making a substantial contribution to HIV prevention among FSWs in Dimapur. However, there was no evidence of improved coverage of HIV prevention services, and there is a clear need to expand the reach of services in order for them to have an impact on a larger pool of FSWs.

## Competing interests

The authors declare that they have no competing interests.

## Authors’ contributions

GA and GKM conceptualized the analysis in this paper. GA undertook the data analysis presented in the paper with inputs from MK, GKM and PG. GA and MK wrote the first draft of the paper. GKM, JM and RSP were involved in designing and coordinating the data collection. RSP, JM and PG provided a strong review and edit of the final draft. All authors read and approved the final manuscript.

## Pre-publication history

The pre-publication history for this paper can be accessed here:

http://www.biomedcentral.com/1471-2458/13/476/prepub
